# Sulfonium Ligands of the α7 nAChR

**DOI:** 10.3390/molecules26185643

**Published:** 2021-09-17

**Authors:** Nicole A. Horenstein, Clare Stokes, Roger L. Papke

**Affiliations:** 1 Department of Chemistry, University of Florida, Gainesville, FL 32611-7200, USA; 2 Department of Pharmacology and Therapeutics, University of Florida, Gainesville, FL 32610-0267, USA; clear@ufl.edu (C.S.); rlpapke@ufl.edu (R.L.P.)

**Keywords:** nicotinic acetylcholine receptor, isostere, sulfonium, desensitize, silent agonist

## Abstract

The α7 nicotinic acetylcholine receptor (nAChR) is an important target given its role in cognitive function as well as in the cholinergic anti-inflammatory pathway, where ligands that are effective at stabilizing desensitized states of the receptor are of particular interest. The typical structural element associated with a good desensitizer is the ammonium pharmacophore, but recent work has identified that a trivalent sulfur, in the positively charged sulfonium form, can substitute for the nitrogen in the ammonium pharmacophore. However, the breadth and scope of employing the sulfonium group is largely unexplored. In this work, we have surveyed a disparate group of sulfonium compounds for their functional activity with α7 as well as other nAChR subtypes. Amongst them, we found that there is a wide range of ability to induce α7 desensitization, with 4-hydroxyphenyldimethylsulfonium and suplatast sulfonium salts being the most desensitizing. The smallest sulfonium compound, trimethylsulfonium, was a partial agonist for α7 and other neuronal nAChR. Molecular docking into the α7 receptor extracellular domain revealed preferred poses in the orthosteric binding site for all but one compound, with typical cation–pi interactions as seen with traditional ammonium compounds. A number of the compounds tested may serve as useful platforms for further development of α7 desensitizing ability and for receptor subtype selectivity.

## 1. Introduction

Nicotinic acetylcholine receptors (nAChR) are a family of pentameric ligand-gated ion channels whose primary physiological agonist is acetylcholine (ACh) [1]. The functional roles of the nAChR are many, ranging from action at the neuromuscular junction, key roles in the central and peripheral nervous system, to modulation of inflammatory responses in leukocytes [2]. The canonical nAChR agonist features an alkylated ammonium group, which bears a positive charge. The minimal pharmacophore to activate a neuronal nAChR is the tetramethylammonium ion (TMA) [3]. Myriad nAChR agonists feature either a quaternary ammonium group or an amine functionality that can be protonated at physiologic pH. A key component of the agonist binding site includes recognition for this positively charged motif via a cation–pi “aromatic box”, [4,5] which utilizes Trp and Tyr residues to provide an electronically complementary site for recognition of the charged ammonium group [6,7,8].

In addition to being notable for its high calcium permeability when activated by ACh and being expressed in brain areas important for cognition, like the hippocampus, the α7 nAChR subtype has also been associated with anti-inflammatory effects via the cholinergic anti-inflammatory pathway (CAP) [2,9]. Ligands that are effective for CAP have been correlated with their ability to induce desensitized states of the α7 receptor; indeed, this particular receptor is known for its susceptibility to rapid desensitization [10]. Two exemplar compounds termed as “silent agonists” for their tendency to desensitize α7 receptors with minimal channel activation are NS6740 and para-trifluoromethyl *N*,*N*-diethyl-*N*′-phenylpiperazinium iodide (*p*-CF_3_ diEPP) compounds [11]. We recently reported synthesis and electrophysiological characterization of a new sulfonium analog of the diethyl-*N*′-phenylpiperazinium scaffold,1-ethyl-4-phenylthiomorpholin-1-ium trifluoromethane sulfonate, in which the quaternary ammonium nitrogen atom is replaced with a sulfur [12]. This compound provides a charge isostere for the ammonium group, yet is not directly analogous given that in the diEPP compound the nitrogen bears two ethyl groups whereas the sulfonium analog has only one. Hence, there are opportunities to place a permanent charge on the sulfur atom that allows a lower level of substitution than is required to quaternize a nitrogen. A broad study of protein data bank (PDB) data for proteins with bound small molecule ligands revealed that the sulfonium group, like the ammonium group, is a preferred chemotype for recognition inside protein aromatic binding cages [13]. There are a limited number of other examples of using a sulfonium group as an isostere for a positively charged nitrogen, or more generally, for a positively charged center. Arecoline is an agonist for muscarinic and some nicotinic receptors [14]. The thio analog of arecoline maintains activity at muscarinic receptors [15]; it has not been tested for nAChR activity. Sulfonium compounds have been used as enzyme inhibitors based on their charge resemblance to high-energy intermediates/transition states, for example, the cationic isoprenoid chemistry of prenyltransferases and squalene synthase [16,17]. Sulfonium analogs of dopamine have been utilized as alternative agonists of the dopamine receptor [18].

**Figure 1:**
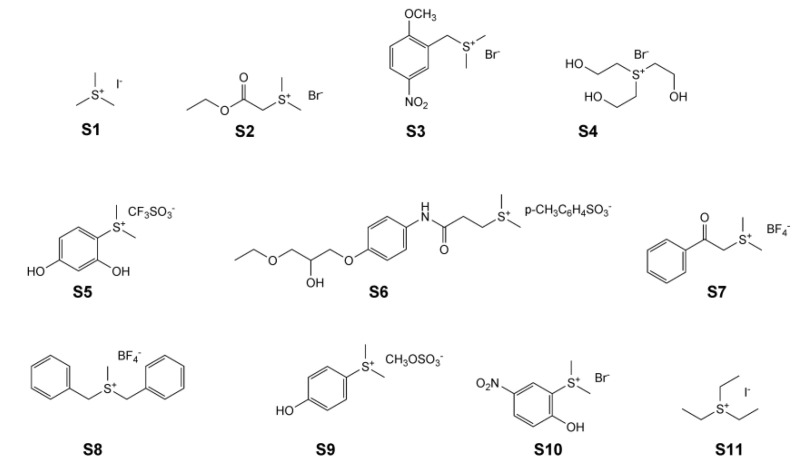
The structures of sulfonium compounds described in this study. Full compound names are defined in Materials and Methods.

In the current study, we conducted a survey of a series of sulfonium compounds, shown in Figure 1, to obtain insights as to how nAChR, particularly α7, responded in terms of activation, desensitization, and antagonism, jointly with the expectation that we would identify some compounds with the desirable properties of silent agonism for the α7 receptor and determine the underlying basis for the activity. Note that **S6** has been previously published as suplatast tosylate and is known to have immunomodulatory activity [19].

## 2. Results

### 2.1. Orthosteric and Silent Agonism of α7

**Figure 2:**
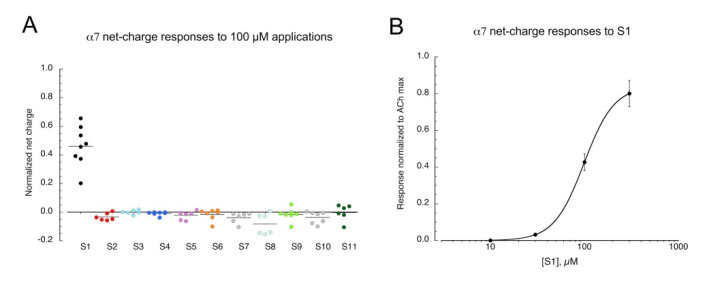
Responses of α7 nAChR to sulfonium compounds. (**A**) Net-charge responses to the applications of the test compounds applied at 100 µM. Net charge was calculated as the integrated area of inward current over a 120 s interval following the 30 s-pre-application period used to establish an initial baseline. Note that points less than zero do not represent outward current responses, per se, but rather reductions in the holding current during the post application period. (**B**) Averaged net-charge responses (±SEM, n = 8) to applications of **S1** across a range of concentrations. Responses were measured relative to preceding 60 µM ACh control responses and then adjusted for the difference between ACh controls and ACh maximum responses. See text for curve-fit values.

The sulfonium compounds (Figure 1) were evaluated for their ability to activate human α7 receptors expressed in *Xenopus* oocytes (Figure 2A). Responses were measured as net charge [20]. **S1**, the smallest of the test compounds, activated receptors, while the other compounds did nothing or produced small reductions in holding currents. The average net-charge response to 100 µM **S1** was 47 ± 6% of the control responses to 60 µM ACh from the same cells. Responses to **S1** across a range of concentrations (n = 8), normalized to the ACh maximum (Figure 2B) were fit to the Hill equation with an I_max_ of 84 ± 0.3% ACh I_max_ and an EC_50_ of 98.8 ± 0.4 µM (Chisq 0.0000028, R = 1).

**Figure 3:**
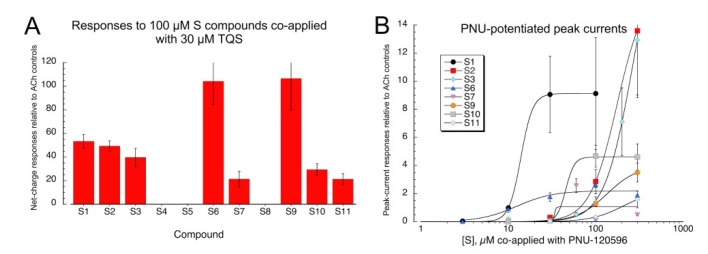
Responses of α7 nAChR to sulfonium compounds coapplied with PAMs. (**A**) Potentiation of α7 responses with 30 µM racemic TQS. Shown are the average net-charge responses of seven or eight oocytes ± SEM. (**B**) Concentration-response studies of peak currents generated by compounds that gave positive responses with TQS, coapplied with 10 µM PNU-120596. Each point represents the average (±SEM) of at least four oocytes. See Table 1 for curve-fit values.

Since we had previously observed that the sulfonium compound, 1-ethyl-4-phenylthiomorpholin-1-ium trifluoromethanesulfonate [12], an analog of the diEPP silent agonists [21], behaved as a silent agonist of α7, inactive when applied alone but active when co-applied with an α7 PAM, we tested the sulfonium compounds in co-application with the type II PAM, 4-naphthalene-1-yl-3a,4,5,9b-tetrahydro-3-*H*-cyclopenta[c]quinoline-8-sulfonic acid amide (TQS) [22]. Compounds were co-applied at a concentration of 100 µM with 30 µM racemic TQS (Figure 3A). All compounds except **S4**, **S5**, and **S8** gave large responses when co-applied with TQS, consistent with silent agonism of α7 (see Table S1 for ANOVA). Active compounds were also evaluated across a range of concentrations with the alternative PAM, PNU-120596 (Figure 3B). The curve-fit values for I_max_ (Table 1) should be taken only as approximation of the relative efficacies of the compounds, due to the large variability frequently observed in PNU-120596-potentiated responses, as evidenced by the large SEMs of the responses obtained with high concentrations. The data may probably be taken as more reliable indicators of the relative potency of the compounds, a feature which may also be influenced by the ionization state of the compounds at physiological pH (see below).

Previous studies of α7 silent agonists [21,23] provided evidence that such agents normally bind to the receptor at sites that are extensions of the orthosteric binding sites for ACh and other efficacious agonists. However, it has also been proposed that some silent agonists may bind to allosteric sites that can couple with the PAMs to produce receptor activation [24].

**Figure 4:**
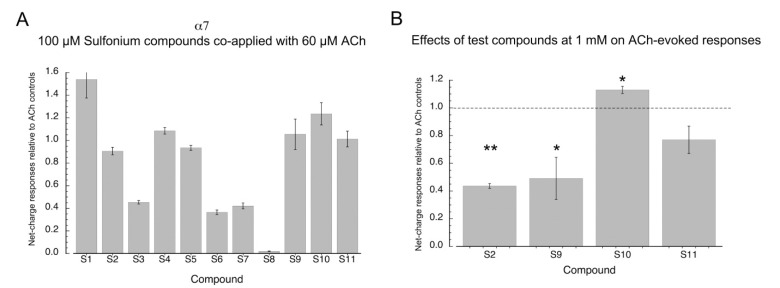
Effects sulfonium compounds on α7 ACh-evoked net-charge responses. (**A**) Effects of the sulfonium compounds at 100 µM co-applied with 60 µM relative to responses to 60 µM ACh applied alone. Data are the averages (±SEM) from seven or eight cells under each condition. See Table S2 for ANOVA. (**B**) Effects of select sulfonium compounds at 1 mM co-applied with 60 µM relative to responses to 60 µM ACh applied alone. Data are the averages (±SEM) from seven or eight cells under each condition. Statistical significance was determined by pairwise *t*-test with *p* values corrected for multiple comparisons; * *p* < 0.05, ** *p* < 0.001.

To determine whether the sulfonium silent agonists functioned by binding to the orthosteric agonist activation sites of α7, we tested how effectively they inhibited responses to 60 µM ACh when co-applied at a concentration of 100 µM (Figure 4A, Table S2 for ANOVA). The effectiveness of the sulfonium compounds at inhibiting the ACh-evoked responses varied greatly, and even for the putative silent agonists, there was no correlation between the potency for evoking PNU-dependent currents and the relative inhibition of ACh responses. Compound **S9**, one of the most potent compounds for activation with PNU-120596 (EC_50_ ≈ 12.5 µM), produced no inhibition of ACh-evoked responses, and **S2** (EC_50_ ≈ 14 µM) produced no more than 10% inhibition at 100 µM. The relatively less potent compounds, **S3** and **S6**, produced about 50% inhibition. It is interesting to note that the compounds **S4** and **S5**, which did not generate responses when co-applied with TQS, also failed to produce inhibition of ACh-evoked responses when co-applied with ACh, suggesting that these two compounds are simply inactive with α7 nAChR.

The sulfonium compounds that were active with TQS but produced little inhibition of ACh response at a concentration of 100 µM were tested as inhibitors of ACh response at a concentration of 1 mM (Figure 4B). Compounds **S2** and **S9** produced significant inhibition under these conditions (*p* < 0.01 and *p* < 0.05, respectively, after correction for multiple comparisons). The effect of **S11** was not statistically significant, and curiously, 1 mM **S10** increased the amplitude of the ACh-evoked responses (*p* < 0.05 after correction for multiple comparisons).

### 2.2. Inhibition of α7 by **S8**

**Figure 5:**
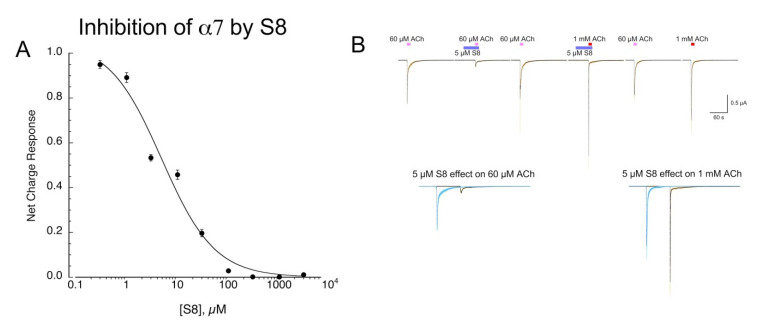
Inhibition of α7 ACh-evoked responses by **S8**. (**A**) Concentration-response studies of α7 net-charge responses to 60 µM ACh co-applied with increasing concentrations of **S8**. To confirm the stability of the ACh responses, applications of ACh plus **S8** were alternated with applications of ACh alone. Data are the averages of eight cells (±SEM). See text for curve-fit values. (**B**) Effects of 5 µM **S8** on responses to either 60 µM or 1 mM ACh. The traces shown across the top are the averaged raw data from seven cells, each normalized to the peak current of the first ACh control response shown. In order to assure that the **S8** was present at 5 µM throughout the ACh-evoked responses, a 30 s pre-application was made of 5 µM **S8** alone. The lower traces show averaged responses to 60 µM or 1 mM ACh alone or with the 5 µM **S8** pre- and co-application. The details of the data are described in Methods.

We tested **S8** across a range of concentrations for its ability to inhibit 60 µM ACh responses (Figure 5A). Note that we use 60 µM as an ACh control because this concentration gives nearly maximal (93%) net-charge responses, with the time course of the responses roughly following the speed of solution exchange [20]. The data were fit with an IC_50_ of 4.9 ± 2.7 µM (Chisq = 0/024, R = 0.989). We tested whether inhibition of α7 responses by **S8** was surmountable by increasing ACh concentration, consistent with a competitive mechanism of inhibition. However, since α7 responses to concentrations of ACh greater than 60 µM occur more rapidly than solution exchange in the chamber [20], we first pre-applied 5 µM **S8** prior to the co-application of 5 µM **S8** with either 60 µM or 1 mM ACh (Figure 5B). The 60 µM net-charge responses were reduced to 28 ± 2% of controls (n = 8) and 60 µM peak currents to 21 ± 3 % controls. The 1 mM net-charge responses were reduced to 56 ± 6 % of those obtained without **S8** co-application, although the **S8** co-application increased the peak-current amplitudes compared to 60 µM controls from 1.78 ± 0.15 to 3.72 ± 0.81 (n = 8). This differential effect of **S8** on the net charge and peak currents of the 1 mM responses suggests that **S8** accelerated the concentration-dependent desensitization of α7 [20].

### 2.3. Sulfonium Compound Effects on Heteromeric nAChR

Concatamers were used in order to obtain heteromeric receptors with known subunit composition. The β2-6-α4 concatamer [25] was co-expressed with monomeric α4, β2, or α5 to obtain receptors with the composition α4(3)β2(2), α4(2)β2(3), or α4(2)β2(2)α5, respectively. A concatamer of five linked subunits [26] was used to obtain the β3α4 β2α6β2 receptors. The α3β4 receptors were formed from the co-expression of α3 and β4 subunit monomers at equal ratios. Note that the data were initially measured relative to internal ACh control responses for each oocyte (Methods). Responses were subsequently adjusted for the relative values of the ACh controls and the ACh maximum responses for each of the various subtypes, determined in previous experiments.

**Figure 6:**
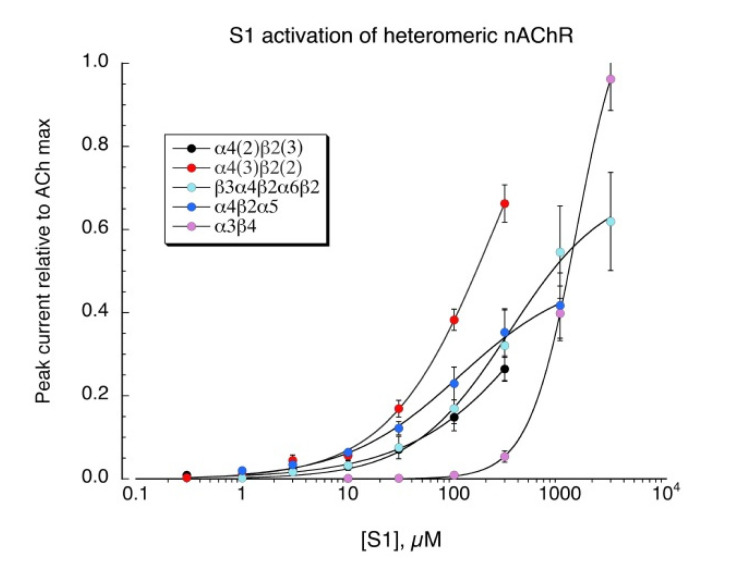
Activation of heteromeric neuronal nAChR by **S1**. Concatamers were used in order to obtain receptors with known subunit composition. The β2-6-α4 concatamer was co-expressed with monomeric α4, β2, or α5 to obtain receptors with the composition α4(3)β2(2), α4(2)β2(3), or α4(2)β2(2)α5, respectively. A concatamer of five linked subunits was used to obtain the β3α4β2 α6β2 receptors. The α3β4 receptors were formed from the co-expression of α3 and β4 subunit monomers at equal ratios. Data are the average peak currents (n = 6–8 oocytes ± SEM), measured relative to initial ACh control responses for each oocyte (concentrations listed in Methods). Responses were subsequently adjusted for the relative values of the ACh controls and the ACh maximum responses for each of the various subtypes, determined in previous experiments. Curve-fit values are provided in Table 2.

**S1** is a structural analog of the minimal pharmacophore for the activation of neuronal nAChR, TMA, and we confirmed that **S1** also activates all the neuronal heteromeric receptors tested (Figure 6, see Table 2 for curve-fit values). Similar to TMA [27], **S1** was not an effective activator of muscle-type (α1β1εδ) receptors (not shown).

**Figure 7:**
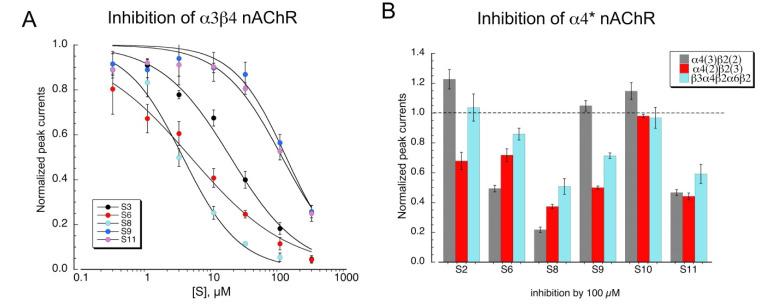
Inhibition of heteromeric nAChR by sulfonium compounds. (**A**) Inhibition of α3β4 100 µM ACh-evoked peak currents by the co-application of the sulfonium compounds. Data are the averaged of six to eight oocytes (±SEM). To confirm the stability of the ACh responses, applications of ACh plus the sulfonium compounds were alternated with applications of ACh alone. Curve-fit values are provided in Table 3. (**B**) Inhibition of ACh control peak-current responses of α4-containing nAChR formed with concatamers by 100 µM of the sulfonium compounds indicated. Data are the average of six to eight oocytes (±SEM).

Although none of the other sulfonium compounds activated neuronal heteromeric receptors, several of them produced inhibition of ACh-evoked responses. Shown in Figure 7A are concentration-response studies for the compounds that were active antagonists of α3β4 nAChR (Table 3). Some compounds were also tested as antagonists of α4β2 subtypes formed with concatamers (Figure 7B). Not surprisingly, **S8** was the most active antagonist, and **S10** was the least.

### 2.4. Silent Agonism of α4β2 Receptors

**Figure 8:**
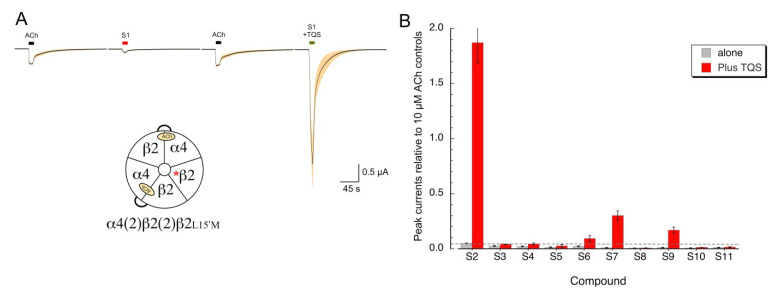
Silent agonism of an α4β2 mutant receptor that is sensitive to the α7 PAM TQS. (**A**) The averaged raw data of responses of eight oocytes expressing the β2-6-α4 concatamer and the β2L15’M mutant to 100 µM of the sulfonium agonist **S1**, applied alone or co-applied with 30 µM racemic TQS. The insert shows a schematic of the subunit configuration predicted for these receptors, with a single mutant β2 subunit outside of the two pairs of α4 and β2 subunits that would form the ACh binding sites. (**B**) Evaluation of the effects of the sulfonium compounds, applied alone at 100 µM or co-applied with 30 µM TQS to α4(2)β2(2)β2L15’M receptors. The dashed line represents our reliable limit of detection for a peak current response relative to application artifacts, which, for these experiments, was 20 nA. Data are the averages of seven to eight oocytes (±SEM), normalized to 10 µM ACh control responses from the same cells.

Our data indicate that several of the sulfonium compounds are α7 silent agonists and appear to be antagonists of alpha4* receptors. We wished to determine whether any of the sulfonium compounds might act as silent agonists on α4β2 receptors, indicating that they promote desensitized states and are not simply antagonists. To test this, we utilized the α4β2L15’M mutant, which makes α4β2 receptors sensitive to the α7 PAM TQS [28], to distinguish between sulfonium compounds that are simple alpha4 antagonists from those that we could classify as silent agonists. As expected, the active agonist **S1** activated the α4β2L15’M mutant receptors (Figure 8A), and that activation was potentiated by TQS. We conducted a screen of the other sulfonium compounds on the α4β2L15’M receptors and identified at least two compounds that gave potentiated responses above our limit of detection indicated by the dotted line in Figure 8B. The relative activity of these potential silent agonists of α4β2 (**S2** >> **S7** > **S9** > **S6**), is a different sequence than what was observed for α7 (**S6** = **S9** > **S2** ≥ **S3** > **S10** = **S9** > **S7**, Figure 3A).

## 3. Discussion

The current study demonstrates the utility and flexibility of a sulfonium as a core element of an alternative nicotinic pharmacophore. The observation that **S1** is a non-selective agonist invites the hypothesis that small elaborations on that structure may lead to other agonists with selectivity for specific nAChR. The identification of several sulfonium silent agonists reveals structural flexibility for that form of α7 receptor modulation.

### Structures and Functions

Compound **S1** can be considered the sulfonium analog of the smallest known agonist for neuronal nAChR, TMA. **S1** bears a permanent positive charge, and by virtue of lacking one methyl group relative to TMA, is also smaller. As expected, **S1** produced potentiated α7 currents when co-applied with TQS or PNU-120596. We can compare compound **S11**, triethylsulfonium, to **S1**. Note that while **S1** is an agonist, **S11** is a silent agonist, only producing α7 activation when combined with a PAM. The structural change between the two compounds is strictly one of size, with the larger **S11** losing agonism but retaining the ability to produce PAM-sensitive desensitization, a trend that has been noted before with simple ammonium compounds [23]. We thought that the acyclic compound **S2** might function as an analog of ACh, but we found it to be a structurally simple and efficacious silent agonist. The basis for lack of agonism in **S2** is unclear, but we note that the distance between the ammonium nitrogen and H-bond accepting carbonyl oxygen of ACh is 5.2 Å, while the corresponding distance in **S2** between the sulfonium sulfur and carbonyl is considerably closer, at 3.8 Å. Compounds **S3**, **S6**, **S7**, **S9**, and **S10** were the remaining ones with silent agonist activity. As noted previously, compound **S6** is suplatast, a known compound with anti-inflammatory activity [19], which has shown silent agonist activity in our present study. It has been proposed that at least the antitussive activity of suplatast may be due to its ability to function as a channel blocker of ganglionic (α3β4) nAChR [29], preventing afferent impulses to muscles associated with cough. Note that, while Zhou et al. reported channel block with 100 µM suplatast, we observed inhibition of α3β4 receptors with an IC_50_ of only 4.5 µM, and our data are consistent with a non-competitive mechanism of inhibition (i.e., channel block) since **S6** inhibition was not surmountable by increasing ACh concentration. Specifically, we observed that 5 µM **S6** was equally effective at inhibiting α3β4 responses to 1 mM ACh as responses to 30 µM ACh (data not shown). However, since suplatast behaves as an α7 silent agonist, and other α7 silent agonists have been shown to effectively modulate CAP, it seems reasonable that the antitussive and other anti-inflammatory activities of suplatast may also be associated with CAP activity.

**Figure 9:**
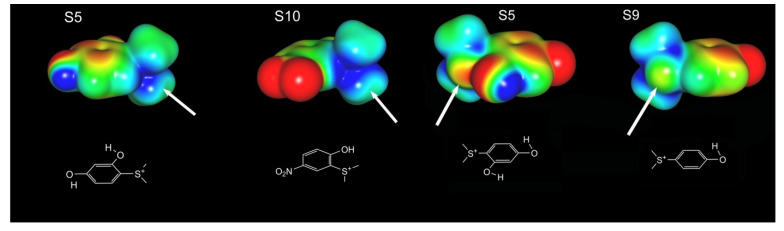
Molecular electrostatic potential (MEP) of compounds **S5**, **S9**, and **S10**. Positive regions are colored blue; negative regions are colored red. The arrow points to the sulfonium sulfur atom. The left panel compares the MEP of **S5** and **S10**; the right panel compares the MEP of **S5** and **S9**. Note that **S5** is presented from two different perspectives in the two figures to best highlight differences between it and either **S9** or **S10**.

One of the questions that emerges from the data is why aromatic sulfoniums as structurally similar as **S5**, **S9**, and **S10** provide different responses when coapplied with TQS. Indeed, compounds **S5** and **S9** virtually overlap in their best docked poses (Supplementary Figure S1), but the α7 response of **S9** and TQS was dramatically greater than that of **S5** and TQS (*p* < 0.001, Table S1). The formation of cation–pi interactions is a key feature of the binding of nicotinic orthosteric ligands, and the electronic distribution of the cation can be compared qualitatively in a series of compounds using molecular electrostatic potentials (MEPs). Analysis of the MEPs shown in Figure 9 reveal that the substituents on the benzene ring can modulate the intensity of the positive charge on the sulfonium center. Compound **S5**, for instance, has two hydroxyl groups which are strongly electron donating, and in comparison to compound **S9**, which has only one hydroxyl group, the intensity of the positive charge is greater. Another factor may be that a lone pair on the ortho hydroxyl substituent is donating electron density through space to the neighboring sulfonium, redistributing its positive charge. Compound **S10** also has an ortho hydroxyl group, but unlike compound **S5**, compound **S10** also has a nitro group, which is a strong electron-withdrawing group that may offset the electron-donating impact of the ortho hydroxyl group. Inspection of the MEP surface shown in Figure 9 does indicate that, relative to **S5**, the **S10** sulfonium bears greater positive electrostatic potential. Given that compounds such as para-nitrophenol are acidic, with a pK_a_ of ~7 for the phenolic hydroxyl, we also considered whether the deprotonated forms of **S5**, **S9**, and **S10** might be accessible at the pH of our experiments. Indeed, titration of **S5** revealed two pK_a_s, one at approximately 6.3, and the other at 9.2 for the second hydroxyl.

Compound **S9** showed an estimated pK_a_ of 7.3, and compound **S10** had a pK_a_ of approximately 5 (data not shown). These data indicate that an electron-deficient sulfonium group conjugated to the phenyl ring can enhance the acidity of a phenolic hydroxyl. Thus, all three compounds have varying proportions of the phenolic OH in the phenoxide form at the pH of Ringers solution used in the experiments. At this time, we cannot rule out that a phenoxide form is active with α7, though conventional wisdom would suggest that activity requires a protonated phenolic group to make the molecule cationic overall, which fits better with the binding model for a nicotinic agonist. Future work on analogs of these compounds may be focused on substituents that are non-ionizable, and thus preserve a positive molecular charge derived from the sulfonium center.

Compound **S7** is interesting in that it may be considered as a phenacyl derivative of the trimethylsulfonium compound **S1**, effectively converting **S1** into a silent agonist. It is also similar to **S2**, in that both are silent, and both contain a carbonyl group in the position beta to the sulfonium sulfur atom. Compound **S7**, containing the phenyl ring, affords a convenient route to further functionalization to enhance its desensitizing properties. The enhanced desensitization observed for some of the aromatic sulfonium compounds, coupled with the wide range of chemistry one can do to functionalize aromatic compounds, argues that they may prove useful as platforms for further development of new α7 nicotinic ligands.

Previous studies have shown the utility of α7 silent agonists as modulators of CAP, and so follow-up studies with these sulfonium silent agonists will be of interest. However, it should be noted that, due to the permanent positive charge carried by these ligands, they are unlikely to cross the blood–brain barrier easily. There is little work reported regarding the pharmacokinetics of sulfonium compounds, but early work on suplatast, **S6**, suggests that distribution to the brain is indeed poor, but is none the less orally bioavailable [30,31]. Suplatast has a considerable amount of chemical functionalization that is remote from the sulfonium center and likely to be influential in oral bioavailability, leading to the idea that other nAChR-active sulfoniums could also be functionalized to control bioavailability.

Silent agonism of α7 receptors can be readily identified through the use of α7 PAMs, and it is hypothesized that these receptors have a metabotropic function that can be activated by such ligands that induce a non-conducting (desensitized) conformation. We have extended our characterization of “silent agonism” through the use of a PAM-sensitive β2 mutant. However, it remains to be determined whether specific functions can be associated with the desensitized states of heteromeric nAChR. One possibility is that α4β2 desensitization may be an approach to modulate the function of these receptors in smokers. However, as noted above, the sulfoniums are unlikely to enter the brain, so it will be of interest to follow up these experiments with evaluation of other ligands for their α4β2 desensitizing activity.

## 4. Materials and Methods

### 4.1. Chemicals and Reagents

Acetylcholine chloride (ACh) and buffer chemicals were purchased from Sigma-Aldrich Chemical Company (St. Louis, MO, USA). PNU-120596 was synthesized in the Horenstein laboratory by Dr. Kinga Chojnacka following the published procedure [32]. TQS was provided by Dr. Ganesh Thakur (Northeastern University, Boston, MA, USA). Sulfonium compounds **S1**–**S4**, **S8**, and **S10** were purchased from Sigma-Aldrich Chemical Company (St. Louis, MO, USA). Sulfonium compounds **S5** and **S6** were purchased from Santa Cruz Biotechnology (Dallas, TX, USA), and sulfonium compounds **S7**, **S9**, and **S11** were purchased from ThermoFisher Scientific (Waltham, MA, USA). The IUPAC names for compounds **S1**–**S11** (Figure 1) are as follows: **S1**, trimethylsulfonium iodide; **S2**, (2-ethoxy-2-oxoethyl)dimethylsulfonium bromide; **S3**, (2-methoxy-5-nitrobenzyl)dimethylsulfonium bromide; **S4**, tris(2-hydroxyethyl)sulfonium bromide; **S5**, (2,4-dihydroxyphenyl)dimethylsulfonium trifluoromethanesulfonate; **S6**, (3-((4-(3-ethoxy-2-hydroxypropoxy)phenyl)amino)-3-oxopropyl)dimethylsulfonium tosylate; **S7**, dimethyl(2-oxo-2-phenylethyl)sulfonium tetrafluoroborate; **S8**, dibenzyl(methyl)sulfonium tetrafluoroborate; **S9**, (4-hydroxyphenyl)dimethylsulfonium methyl sulfate; **S10**, (2-hydroxy-5-nitrophenyl)dimethylsulfonium bromide; **S11**, triethylsulfonium iodide.

### 4.2. Expression in Xenopus Oocytes

The human nAChR clones were obtained from Jon Lindstrom (University of Pennsylvania, Philadelphia, PA, USA). Mouse muscle subunit clones were obtained from Jim Boulter (Salk Institute, La Jolla, CA, USA) and Paul Gardner (Dartmouth, Hanover, NH, USA). The human resistance-to-cholinesterase 3 (RIC3) clone was obtained from Millet Treinin (Hebrew University, Jerusalem, Israel) and co-injected with α7 to improve the level and speed of α7 receptor expression without affecting the pharmacological properties of the receptors [33]. Subsequent to linearization and purification of the plasmid DNAs, RNAs were prepared using the mMessage mMachine in vitro RNA transcription kit (Ambion, Austin, TX, USA).

Oocytes were surgically removed from mature female *Xenopus laevis* frogs (Nasco, Ft. Atkinson, WI, USA) as previously described [34]. Frogs were maintained in the Animal Care Service facility of the University of Florida, and all procedures were approved by the University of Florida Institutional Animal Care and Use Committee (approval number 202002669).

### 4.3. Two-Electrode Voltage Clamp Electrophysiology

Experiments were conducted using OpusXpress 6000A (Molecular Devices, Union City, CA, USA) [34]. Both the voltage and current electrodes were filled with 3 M KCl. Oocytes were voltage-clamped at −60 mV at room temperature (24 °C). The oocytes were bath-perfused with Ringer’s solution (115 mM NaCl, 2.5 mM KCl, 1.8 mM CaCl_2_, 10 mM HEPES, and 1 μM atropine, pH 7.2) at 2 mL/min for α7 receptors, or 4 mL/min for heteromeric nAChR. To evaluate the effects of experimental compounds, responses were compared to control ACh-evoked responses, defined as the average of two initial applications of ACh made before test applications. ACh control concentrations were 60 µM for α7 receptors; 100 µM for α4(3)β2(2) and α3β4 receptors; 30 µM for α4β2α6β2β3 and muscle-type (αβ1εδ) receptors; and 10 µM for α4(3)β2(2) and α4(2)β2(2)α5 receptors.

Solutions were applied from 96-well plates via disposable tips. Drug applications were 12 s in duration, followed by 181 s washout periods for α7 receptors, or 6 s in duration followed by 301 s washout periods for heteromeric nAChR. The responses were calculated as both peak current amplitudes and net charge, as previously described [20]. Data were collected at 50 Hz, filtered at 20 Hz for α7 receptors or filtered at 5 Hz for heteromeric nAChR, and analyzed by Clampfit (Molecular Devices, San Jose, CA, USA) and Excel (Microsoft, Redmond, WA, USA). Data were expressed as means ± SEM from at least four oocytes for each experiment and plotted with Kaleidagraph 4.5.2 (Abelbeck Software, Reading, PA, USA). Multi-cell averages were calculated for comparisons of complex responses. Averages of the normalized data were calculated for each of the 10,322 points in each of the 206.44 s traces (acquired at 50 Hz), as well as the standard errors for those averages.

### 4.4. Data and Statistical Analysis

Comparisons of results were made using one-way ANOVA or using *t*-tests between the pairs of experimental measurements. In cases where multiple comparisons were made, a Bonferroni correction for multiple comparisons [35] was applied to correct for possible false positives. A value of *p* < 0.05 was used to constitute a minimum level of significance. The statistics were calculated using an Excel template provided in Microsoft Office, or ANOVA protocols in Kaleidagraph (4.5.2 Abelbeck Software, Reading, PA, USA).

### 4.5. Computational Work

The recent corrected structure for the α7 nAChR was employed for the docking studies (PDB ID 7KOX). The extracellular domain from two neighboring subunits was extracted for docking with Glide (Schrodinger, New York, NY, USA). Docking grids were set to encompass both the orthosteric site and the allosteric activation site in the vestibule [36]. Glide docking employed XP mode [37,38,39]. Gaussian 09 [40] was used to minimize the structure of sulfonium ligands at the Restricted Hartree–Fock level of theory and 6-31G* basis set. Cube data for electron density and molecular electrostatic potential were extracted from Gaussian checkpoint files and visualized with GabEdit 2.5.0 [41], in which the MEP data were mapped onto a surface at a level of 0.002 e-isodensity, and color-coded from most negative (red) to most positive (blue).

## Figures and Tables

**Table 1:** Parameters extracted from curve fits for Figure 3B ^1^.

PNU Activation	I_Max_	EC_50_	Chisq	R
**S1**	9.1 ± 0.05	14 ± 0.7	0.0025	0.9998
**S2**	16 ± 6	175 ± 79	0.03	0.998
**S3**	22 ± 2.5	263 ± 23	0.07	0.9997
**S6**	2.2 ± 0.3	12.5 ± 4.9	0.33	0.957
**S7**	>1.1	N.A. ^1^	N.A.	N.A.
**S9**	3.98 ± 0.14	135 ± 6.7	0.0005	0.99997
**S10**	4.6 ± 0.1	45.5 ± 50.6	0.008	0.9998
**S11**	>1.8	N.A.	N.A.	N.A.

^1^ Average responses were fit to the Hill equation by the Levenberg-Marquardt algorithm in Kaleidagraph. Error estimates on the fit parameters were calculated based on that analysis and reflect the goodness of fit to the Hill equation, as reflected in the chi sq value and correlation co-efficient. N.A. not available since these data were not able to be fit to the Hill equation.

**Table 2:** Parameters extracted from curve fits for Figure 6.

S1 Activation	I_Max_	EC_50_	Chisq	R
α7	0.84 ± 0.01	98 ± 0.5	0.00003	1
α4(2)β2(3)	0.89 ± 0.44	1060 ± 1240	0.0005	0.991
α4(3)β2(2)	1.2 ± 0.2	246 ± 104	0.005	0.9992
β3α4 β2α6β2	0.71 ± 0.35	333 ± 51	0.001	0.998
α4β2α5	0.50 ± 0.03	114 ± 25	0.0004	0.999
α3β4	1.18 ± 0.01	1417 ± 19.4	0.000008	0.99999

**Table 3:** Parameters extracted from curve fits for Figure 7A.

α3β4 Inhibition	IC_50_	Chisq	R
**S3**	18 ± 2.6	0.13	0.9917
**S6**	4.5 ± 0.6	0.006	0.994
**S8**	3.4 ± 0.4	0.006	0.995
**S9**	124 ± 22	0.024	0.97
**S11**	108 ± 20	0.21	0.97

## Data Availability

Data is contained within the article or Supplementary Materials. The data presented in this study are available in this article.

## Supplementary Materials

The following are available online. Table S1 provides statistical analysis for data presented in Figure 3. Table S2 provides statistical analysis for data presented in Figure 4. Figure S1 presents docked poses for compounds **S5** and **S9**.

## Sample Availability

Samples of the compounds are not available from the authors.
