# Supplementary material for: Sulfonium Ligands of the α7 nAChR
Source: Molecules. 2021 Sep 17;26(18):5643. doi: 10.3390/molecules26185643 (PMC8464850; doi:10.3390/molecules26185643)
Supplement: Supplementary file 1 [file molecules-26-05643-s001.zip › Figure S1.pdf]

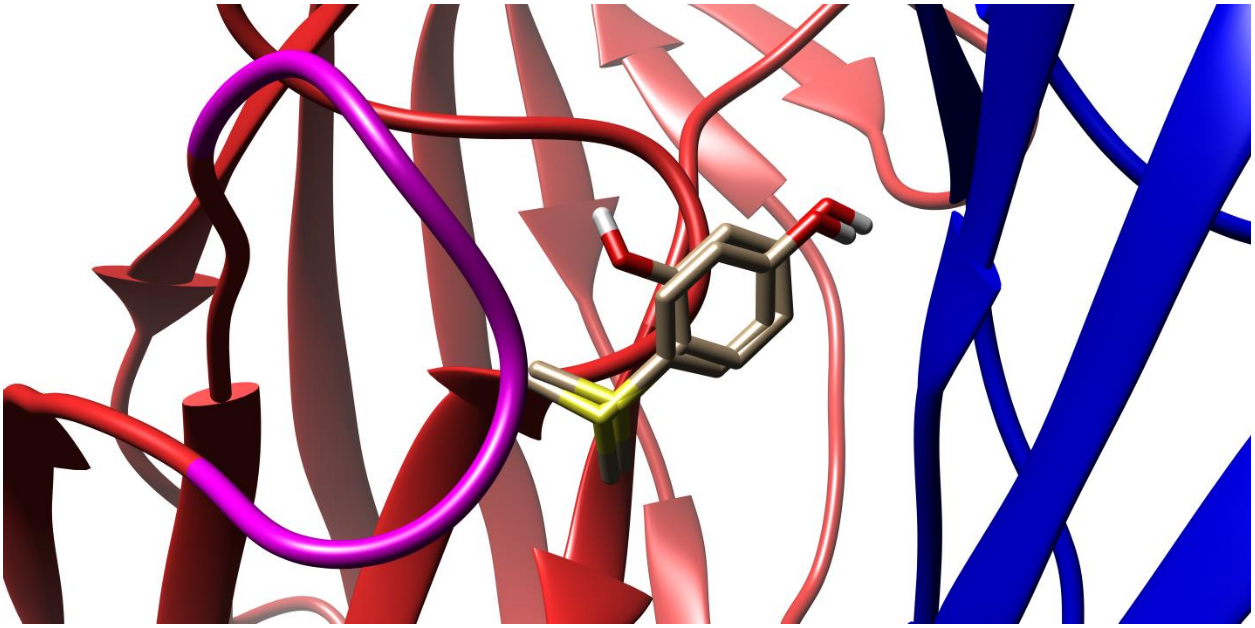

Figure S1. Best poses for docking compounds S5 and S9 into α7. The red ribbon corresponds to the (+) side of the subunit interface and the blue ribbon corresponds to the (-) side of the subunit interface. The C-loop, covering the orthosteric site is highlighted in magenta.
