# Supplementary material for: Sulfonium Ligands of the α7 nAChR
Source: Molecules. 2021 Sep 17;26(18):5643. doi: 10.3390/molecules26185643 (PMC8464850; doi:10.3390/molecules26185643)
Supplement: Supplementary file 1 [file molecules-26-05643-s001.zip › Table S1rev.pdf]

**Table S1.** Statistical analysis for data of Figure 3. One Way ANOVA Figure 3A
Factor A: 11 Groups
S1, S2, S3, S4, S5, S6, S7, S8, S9, S10, S11

Analysis of Variance Results

| Source | DF | SS | MS | F | P |
|---|---|---|---|---|---|
| Total | 85 | 186788.76 | 2197.5148 | | |
| A | 10 | 114990.43 | 11499.043 | 12.011814 | < 0.0001 |
| Error | 75 | 71798.333 | 957.31111 | | |

Bonferroni's All Pairs Comparison

| Comparison | Mean Difference | \|t\| | P | 95% CL |
|---|---|---|---|---|
| S1 vs S2 | 4.07286 | 0.2543 | 1 | -51.252 to 59.397 |
| S1 vs S3 | 13.6674 | 0.8264 | 1 | -43.472 to 70.806 |
| S1 vs S4 | 53.3732 | 3.3331 | 0.0735 | -1.9514 to 108.7 |
| S1 vs S5 | 53.2813 | 3.3273 | 0.0748 | -2.0433 to 108.61 |
| S1 vs S6 | -50.7983 | 3.1723 | 0.1206 | -106.12 to 4.5263 |
| S1 vs S7 | 32.0249 | 1.9999 | 1 | -23.3 to 87.35 |
| S1 vs S8 | 53.4841 | 3.34 | 0.0719 | -1.8406 to 108.81 |
| S1 vs S9 | -53.1547 | 3.3194 | 0.0767 | -108.48 to 2.17 |
| S1 vs S10 | 24.0136 | 1.4996 | 1 | -31.311 to 79.338 |
| S1 vs S11 | 32.0732 | 2.0029 | 1 | -23.251 to 87.398 |
| S2 vs S3 | 9.59449 | 0.5992 | 1 | -45.73 to 64.919 |
| S2 vs S4 | 49.3004 | 3.1868 | 0.1154 | -4.1483 to 102.75 |
| S2 vs S5 | 49.2085 | 3.1809 | 0.1175 | -4.2402 to 102.66 |
| S2 vs S6 | -54.8712 | 3.5469 | 0.0372 | -108.32 to -1.4225 |
| S2 vs S7 | 27.952 | 1.8068 | 1 | -25.497 to 81.401 |
| S2 vs S8 | 49.4112 | 3.194 | 0.1129 | -4.0375 to 102.86 |
| S2 vs S9 | -57.2275 | 3.6992 | 0.0225 | -110.68 to -3.7789 |
| S2 vs S10 | 19.9407 | 1.289 | 1 | -33.508 to 73.389 |
| S2 vs S11 | 28.0004 | 1.81 | 1 | -25.448 to 81.449 |
| S3 vs S4 | 39.7059 | 2.4796 | 0.8469 | -15.619 to 95.03 |
| S3 vs S5 | 39.614 | 2.4738 | 0.8596 | -15.711 to 94.939 |
| S3 vs S6 | -64.4657 | 4.0258 | 0.0074 | -119.79 to -9.1411 |
| S3 vs S7 | 18.3575 | 1.1464 | 1 | -36.967 to 73.682 |
| S3 vs S8 | 39.8167 | 2.4865 | 0.8319 | -15.508 to 95.141 |
| S3 vs S9 | -66.822 | 4.1729 | 0.0044 | -122.15 to -11.497 |
| S3 vs S10 | 10.3462 | 0.6461 | 1 | -44.978 to 65.671 |
| S3 vs S11 | 18.4059 | 1.1494 | 1 | -36.919 to 73.73 |
| S4 vs S5 | -0.0918904 | 0.0059 | 1 | -53.541 to 53.357 |
| S4 vs S6 | -104.172 | 6.7337 | < 0.0001 | -157.62 to -50.723 |
| S4 vs S7 | -21.3483 | 1.38 | 1 | -74.797 to 32.1 |
| S4 vs S8 | 0.110824 | 0.0072 | 1 | -53.338 to 53.559 |
| S4 vs S9 | -106.528 | 6.886 | < 0.0001 | -159.98 to -53.079 |
| S4 vs S10 | -29.3597 | 1.8978 | 1 | -82.808 to 24.089 |
| S4 vs S11 | -21.3 | 1.3768 | 1 | -74.749 to 32.149 |

| | | | | |
|---|---|---|---|---|
| S5 vs S6 | -104.08 | 6.7277 | < 0.0001 | -157.53 to -50.631 |
| S5 vs S7 | -21.2564 | 1.374 | 1 | -74.705 to 32.192 |
| S5 vs S8 | 0.202714 | 0.0131 | 1 | -53.246 to 53.651 |
| S5 vs S9 | -106.436 | 6.8801 | < 0.0001 | -159.88 to -52.987 |
| S5 vs S10 | -29.2678 | 1.8919 | 1 | -82.716 to 24.181 |
| S5 vs S11 | -21.2081 | 1.3709 | 1 | -74.657 to 32.241 |
| S6 vs S7 | 82.8232 | 5.3537 | < 0.0001 | 29.375 to 136.27 |
| S6 vs S8 | 104.282 | 6.7408 | < 0.0001 | 50.834 to 157.73 |
| S6 vs S9 | -2.35634 | 0.1523 | 1 | -55.805 to 51.092 |
| S6 vs S10 | 74.8119 | 4.8359 | 0.0004 | 21.363 to 128.26 |
| S6 vs S11 | 82.8715 | 5.3568 | < 0.0001 | 29.423 to 136.32 |
| S7 vs S8 | 21.4592 | 1.3871 | 1 | -31.989 to 74.908 |
| S7 vs S9 | -85.1796 | 5.506 | < 0.0001 | -138.63 to -31.731 |
| S7 vs S10 | -8.01134 | 0.5179 | 1 | -61.46 to 45.437 |
| S7 vs S11 | 0.0483237 | 0.0031 | 1 | -53.4 to 53.497 |
| S8 vs S9 | -106.639 | 6.8932 | < 0.0001 | -160.09 to -53.19 |
| S8 vs S10 | -29.4705 | 1.905 | 1 | -82.919 to 23.978 |
| S8 vs S11 | -21.4108 | 1.384 | 1 | -74.859 to 32.038 |
| S9 vs S10 | 77.1682 | 4.9882 | 0.0002 | 23.72 to 130.62 |
| S9 vs S11 | 85.2279 | 5.5092 | < 0.0001 | 31.779 to 138.68 |
| S10 vs S11 | 8.05967 | 0.521 | 1 | -45.389 to 61.508 |

DF; degrees of freedom
SS; sum of squares
MS; mean square
F; F statistic
P; probability
|t|;t value
CL; confidence limit
