# Supplementary material for: Sulfonium Ligands of the α7 nAChR
Source: Molecules. 2021 Sep 17;26(18):5643. doi: 10.3390/molecules26185643 (PMC8464850; doi:10.3390/molecules26185643)
Supplement: Supplementary file 1 [file molecules-26-05643-s001.zip › Table S2rev.pdf]

Table S2. Statistical analysis for data of Figure 4. One Way ANOVA Figure 4A

Data Table:

Factor A: 11 Groups

S1, S2, S3, S4, S5, S6, S7, S8, S9, S10, S11

Analysis of Variance Results

Source	DF	SS	MS	F	P
Total	84	19.395502	0.23089883		
A	10	15.891665	1.5891665	33.56273	< 0.0001
Error	74	3.5038366	0.047349144		

Bonferroni's All Pairs Comparison

Comparison	Mean Difference	|t|	P	95% CL
S1 vs S2	0.633329	5.6237	< 0.0001	0.24402 to 1.0226
S1 vs S3	1.08606	9.9822	< 0.0001	0.70995 to 1.4622
S1 vs S4	0.454079	4.1735	0.0044	0.077974 to 0.83018
S1 vs S5	0.604589	5.3685	< 0.0001	0.21528 to 0.99389
S1 vs S6	1.17457	10.4297	< 0.0001	0.78526 to 1.5639
S1 vs S7	1.11838	10.2793	< 0.0001	0.74227 to 1.4945
S1 vs S8	1.51958	13.9669	< 0.0001	1.1435 to 1.8957
S1 vs S9	0.484814	4.456	0.0016	0.10871 to 0.86092
S1 vs S10	0.303266	2.7874	0.3712	-0.072839 to 0.67937
S1 vs S11	0.526359	4.8379	0.0004	0.15025 to 0.90246
S2 vs S3	0.452729	4.02	0.0076	0.063424 to 0.84203
S2 vs S4	-0.17925	1.5917	1	-0.56856 to 0.21006
S2 vs S5	-0.0287402	0.2471	1	-0.43081 to 0.37333
S2 vs S6	0.54124	4.6534	0.0008	0.13917 to 0.94331
S2 vs S7	0.48505	4.307	0.0028	0.095744 to 0.87436
S2 vs S8	0.886256	7.8696	< .0001	0.49695 to 1.2756
S2 vs S9	-0.148515	1.3188	1	-0.53782 to 0.24079
S2 vs S10	-0.330063	2.9308	0.2472	-0.71937 to 0.059243
S2 vs S11	-0.10697	0.9498	1	-0.49628 to 0.28234
S3 vs S4	-0.631979	5.8087	< 0.0001	-1.0081 to -0.25587
S3 vs S5	-0.48147	4.2752	0.0031	-0.87078 to -0.092164
S3 vs S6	0.0885109	0.7859	1	-0.30079 to 0.47782
S3 vs S7	0.0323203	0.2971	1	-0.34378 to 0.40843
S3 vs S8	0.433526	3.9846	0.0086	0.057421 to 0.80963
S3 vs S9	-0.601245	5.5262	< 0.0001	-0.97735 to -0.22514
S3 vs S10	-0.782792	7.1948	< 0.0001	-1.1589 to -0.40669
S3 vs S11	-0.559699	5.1443	0.0001	-0.9358 to -0.18359
S4 vs S5	0.15051	1.3365	1	-0.2388 to 0.53982
S4 vs S6	0.72049	6.3976	< 0.0001	0.33118 to 1.1098
S4 vs S7	0.6643	6.1057	< 0.0001	0.28819 to 1.0404
S4 vs S8	1.06551	9.7933	< 0.0001	0.6894 to 1.4416
S4 vs S9	0.0307346	0.2825	1	-0.34537 to 0.40684
S4 vs S10	-0.150813	1.3862	1	-0.52692 to 0.22529

S4 vs S11	0.0722799	0.6643	1	-0.30382 to 0.44838
S5 vs S6	0.569981	4.9005	0.0003	0.16791 to 0.97205
S5 vs S7	0.51379	4.5622	0.0011	0.12448 to 0.9031
S5 vs S8	0.914996	8.1248	< 0.0001	0.52569 to 1.3043
S5 vs S9	-0.119775	1.0636	1	-0.50908 to 0.26953
S5 vs S10	-0.301323	2.6756	0.5048	-0.69063 to 0.087983
S5 vs S11	-0.0782297	0.6946	1	-0.46754 to 0.31108
S6 vs S7	-0.0561906	0.4989	1	-0.4455 to 0.33311
S6 vs S8	0.345015	3.0636	0.1677	-0.04429 to 0.73432
S6 vs S9	-0.689756	6.1247	< 0.0001	-1.0791 to -0.30045
S6 vs S10	-0.871303	7.7368	< 0.0001	-1.2606 to -0.482
S6 vs S11	-0.64821	5.7558	< 0.0001	-1.0375 to -0.2589
S7 vs S8	0.401206	3.6876	0.0236	0.025101 to 0.77731
S7 vs S9	-0.633565	5.8232	< 0.0001	-1.0097 to -0.25746
S7 vs S10	-0.815113	7.4919	< 0.0001	-1.1912 to -0.43901
S7 vs S11	-0.59202	5.4414	< 0.0001	-0.96812 to -0.21591
S8 vs S9	-1.03477	9.5108	< 0.0001	-1.4109 to -0.65867
S8 vs S10	-1.21632	11.1795	< 0.0001	-1.5924 to -0.84021
S8 vs S11	-0.993226	9.129	< 0.0001	-1.3693 to -0.61712
S9 vs S10	-0.181548	1.6686	1	-0.55765 to 0.19456
S9 vs S11	0.0415453	0.3819	1	-0.33456 to 0.41765
S10 vs S11	0.223093	2.0505	1	-0.15301 to 0.5992

DF; degrees of freedom
SS; sum of squares
MS; mean square
F; F statistic
P; probability
|t|;t value
CL; confidence limit
